# Lifelong cerebrovascular disease burden among CADASIL patients: analysis from a global health research network

**DOI:** 10.3389/fneur.2023.1203985

**Published:** 2023-07-14

**Authors:** Alan P. Pan, Thomas Potter, Abdulaziz Bako, Jonika Tannous, Sudha Seshadri, Louise D. McCullough, Farhaan S. Vahidy

**Affiliations:** ^1^Center for Health Data Science and Analytics, Houston Methodist, Houston, TX, United States; ^2^Department of Neurosurgery, Houston Methodist, Houston, TX, United States; ^3^Glenn Biggs Institute for Alzheimer’s and Neurodegenerative Diseases, University of Texas Health Science Center, San Antonio, TX, United States; ^4^Department of Neurology, McGovern Medical School, University of Texas Health Science Center, Houston, TX, United States; ^5^Department of Population Health Sciences, Weill Cornell Medicine, New York, NY, United States

**Keywords:** CADASIL, vascular malformation, stroke prevalence, sex differences, risk factors

## Abstract

**Introduction:**

Data reporting on patients with Cerebral Autosomal Dominant Arteriopathy with Subcortical Infarcts and Leukoencephalopathy (CADASIL) within the United States population is limited. We sought to evaluate the overt cerebrovascular disease burden among patients with CADASIL.

**Methods:**

Harmonized electronic medical records were extracted from the TriNetX global health research network. CADASIL patients were identified using diagnostic codes and those with/without history of documented stroke sub-types (ischemic stroke [IS], intracerebral hemorrhage [ICH], subarachnoid hemorrhage [SAH] and transient ischemic attack [TIA]) were compared. Adjusted odds ratios (OR) and 95% confidence intervals (CI) of stroke incidence and mortality associated with sex were computed.

**Results:**

Between September 2018 and April 2020, 914 CADASIL patients were identified (median [IQR] age: 60 [50–69], 61.3% females); of whom 596 (65.2%) had documented cerebrovascular events (i.e., CADASIL-Stroke patients). Among CADASIL-Stroke patients, 89.4% experienced an IS, co-existing with TIAs in 27.7% and hemorrhagic strokes in 6.2%; initial stroke events occurred ≤65 years of age in 71% of patients. CADASIL-Stroke patients (vs. CADASIL-non-Stroke) had higher cardiovascular and neurological (migraines, cognitive impairment, epilepsy/seizures, mood disorders) burden. In age- and comorbidity-adjusted models, males had higher associated risk of stroke onset (OR: 1.37, CI: 1.01–1.86). Mortality risk was higher for males (OR: 2.72, CI: 1.53–4.84).

**Discussion:**

Early screening and targeted treatment strategies are warranted to help CADASIL patients with symptom management and risk mitigation.

## Introduction

Cerebral Autosomal Dominant Arteriopathy with Subcortical Infarcts and L eukoencephalopathy (CADASIL) is the most common and well characterized genetically inherited cause of cerebral small vessel disease (cSVD). CADASIL is caused by mutations in the *NOTCH3* gene which result in abnormal smooth muscle protein formation, primarily in the cerebral vasculature (1–4). CADASIL has an estimated prevalence between 2 and 5 cases per 100,000 individuals (5–9) and is associated with multiple neurological and neuro-psychiatric symptoms (10, 11). The primary causes of CADASIL-associated morbidity and mortality are represented by early onset stroke and vascular cognitive impairment (12, 13).

Most observational investigations on CADASIL have focused on patient populations in Europe or Asia, with fewer studies from the United States (5–8, 14). As such, there is a need to accurately report on the prevalence of CADASIL and further study the disease etiology and associated clinical manifestations. Furthermore, given accumulating evidence suggesting sex differences in cSVD (15, 16), validation within United States cohorts is necessary. Based on shared data from a harmonized clinical research repository, we evaluated the overt cerebrovascular disease burden in a large sample of patients with CADASIL in the United States.

## Methods

### Study setting and design

We accessed harmonized and de-identified electronic medical record (EMR) data from the TriNetX global health research network (17). Available data span across hospital, primary care, and specialty treatment encounters and include information on demographics, diagnoses, medications, procedures, vital sign measurements, and outcomes. Data requested for the study were received from the source registry on April 16, 2021. The ‘Strengthening the Reporting of Observational Studies in Epidemiology’ (STROBE) reporting guideline was followed for this study.

### Primary outcomes and measures

Patients with CADASIL were identified by querying diagnosis records for International Classification of Diseases, Tenth Revision (ICD-10) codes [I67.850]. CADASIL patients with and without documented history of cerebrovascular event sub-types (ischemic stroke [IS], intracerebral hemorrhage [ICH], subarachnoid hemorrhage [SAH], transient ischemic attack [TIA]) were flagged using ICD-9 and ICD-10 codes: IS (433; I63), ICH (431; I61), SAH (430; I60), TIA (435.9; G45.9).

Using diagnostic codes (ICD-10), diagnoses and events of co-existing neurological disorders and cerebrovascular diseases were captured from patient records, including migraines, cognitive impairment and dementia, neuro-psychiatric symptoms, epilepsy, and recurrent seizures. The age at first stroke and/or death was derived for patients with this information available as well as the timing of stroke events in relation to formal diagnosis of CADASIL. Available information on level of impairment and consciousness – National Institutes of Health Stroke Scale (NIHSS) [ICD-10: R29.7x] and Glasgow Coma Scale (GCS) [ICD-10: R40.24x] – were also abstracted from patient records.

### Other covariates

Co-existing conditions and overall disease burden (as quantified by the Charlson Comorbidity Index [CCI] score) were also determined. Other variables of interest include demographic factors (age, sex, race, ethnicity), medication administration, procedures, and vital sign measurements (body mass index [BMI], systolic [SBP] and diastolic blood pressure [DBP], temperature, respiratory rate, heart rate). De-identified, three-digit patient ZIP Codes (0XX – 9XX) were abstracted to report prevalence of CADSIL across various United States Census divisions.

### Statistical analyses

Descriptive statistics (means, standard deviations, interquartile ranges [IQR], proportions) and group difference tests (Wilcoxon rank-sum, Chi-squared) were performed to report bivariable comparisons of demographics, comorbidities, medications, vital signs, and outcomes between CADASIL patients with and without history of cerebrovascular events (CADASIL-Stroke vs. CADASIL-non-Stroke). Sex-stratified analyses were also conducted for the overall CADASIL cohort. Logistic regression models were fit to estimate the age-adjusted odds of stroke diagnosis and mortality associated with sex. Using these model estimates, we further derived and depicted the sex-stratified probabilities of stroke onset and mortality across the spectrum of age. Kaplan–Meier curves for sex-stratified cumulative incidences of stroke onset and mortality were also depicted and compared using log-rank tests. Odds ratios (ORs) and 95% confidence intervals (95% CI) are reported. Missingness of data are reported in the tables and results. Analyses were performed using R statistical software (The R Foundation; version 4.0.5).

### Standard protocol approvals, registrations, and patient consents

This study uses de-identified shared data from the TriNetX global health research network. The TriNetX Analytics network is Health Insurance Portability and Accountability Act (HIPAA) compliant and all healthcare organization partners contribute data under business associate agreements which assert that all approvals and consents have been met to share data for research purposes.

## Results

Between September 24th, 2018 and April 17th, 2020, 914 patients diagnosed with CADASIL were identified, of whom 596 (65.2%) had documented diagnosis of a stroke sub-type (Supplementary Table S1). The median (IQR) age of CADASIL patients at their last recorded clinical encounter was 60 (50–69) years, and females comprised of 61.3% of the overall cohort. CADASIL patients in the East South Central (12.5%), Pacific (12.5%), Middle Atlantic (11.5%), and East North Central (11.3%) regions of the United States accounted for the highest relative proportions of all reported cases. Further descriptive characteristics of the overall CADASIL cohort as well as those with and without history of cerebrovascular events are provided in Supplementary Table S1.

### Differences in CADASIL patient cohort characteristics by history of cerebrovascular event

**Figure 1:**
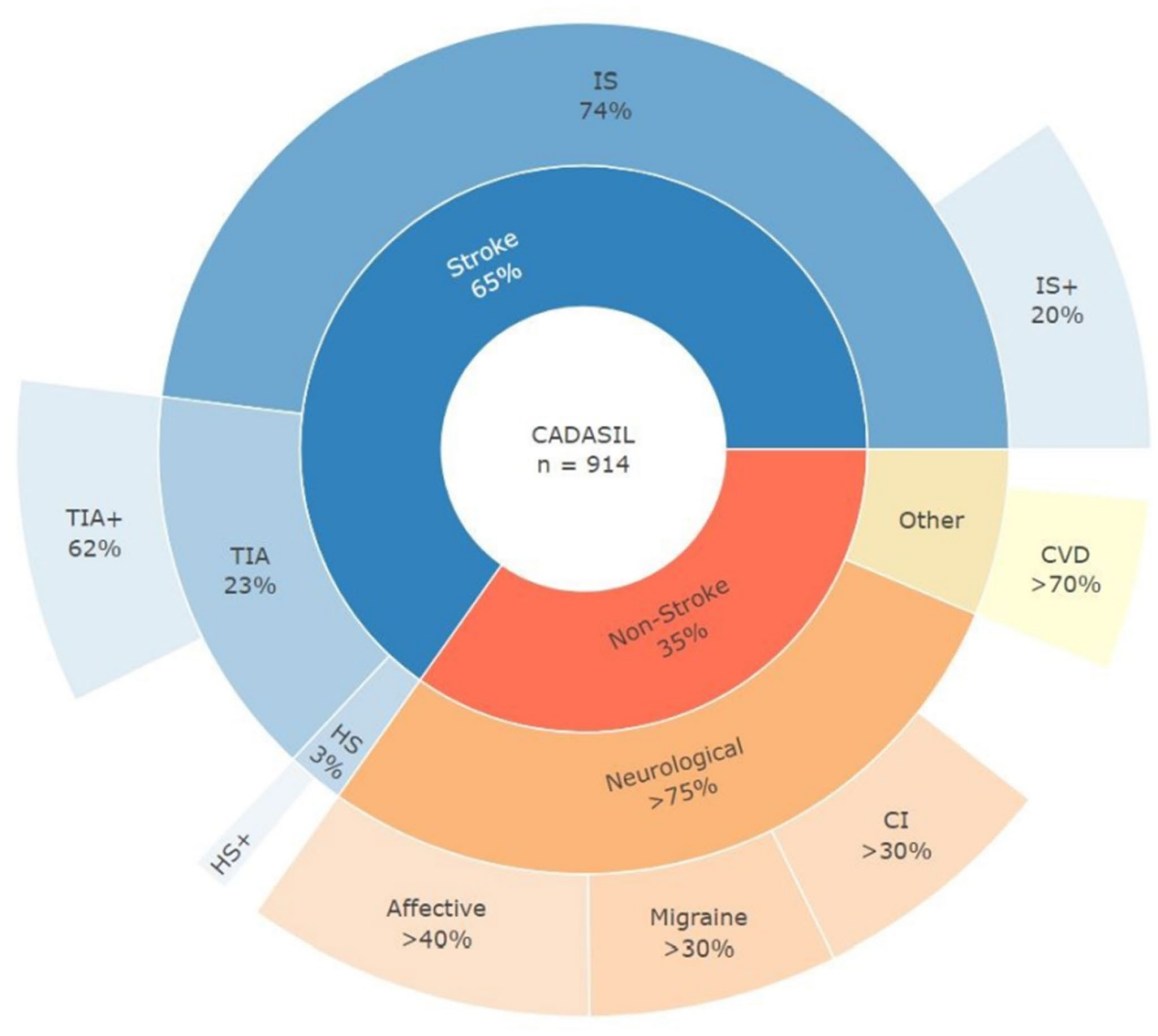
Clinical presentation and diagnoses in CADASIL patients. IS, initial ischemic stroke event; TIA, initial transient ischemic attack event; HS, initial hemorrhagic stroke event; IS+, IS and other secondary stroke; TIA+, TIA and other secondary stroke; HS+, HS and other secondary stroke; CI, cognitive impairment; CVD, cardiovascular disease.

Figure 1 represents various clinical presentations and co-existing diagnoses among CADASIL patients with and without history of cerebrovascular event. Among all CADASIL-Stroke patients, 89.4% had at least one documented IS, 27.7% had at least one TIA, and 6.2% had a history of a hemorrhagic stroke. The initial stroke event occurred before 50 years of age in 30% of CADASIL-Stroke patients and before 65 years in 71% of patients.

CADASIL-Stroke patients were older as of their last recorded clinical encounter, compared to CADASIL-non-Stroke patients (median [IQR] age in years: 61 [51–69] vs. 59 [48–69]; *p* = 0.044). In addition to a higher burden of cardiovascular risk factors – hypertension (70.1% vs. 60.4%; *p* = 0.004), atrial fibrillation (10.2% vs. 5.7%; *p* = 0.026), hyperlipidemia (78.0% vs. 57.5%; *p* < 0.001), peripheral vascular disease (36.2% vs. 23.6%; *p* < 0.001), diabetes (32.4% vs. 24.8%; *p* = 0.022) – a higher proportion of CADASIL-Stroke patients (vs. CADASIL-non-Stroke) were diagnosed with other neurological conditions, including migraines (36.7% vs. 29.9%; *p* = 0.044), cognitive impairment (38.8% vs. 24.2%; *p* < 0.001), epilepsy / seizures (18.6% vs. 11.6%; *p* = 0.008), and mood disorders (52.9% vs. 40.9%; *p* = 0.001). Overall disease burden was higher for CADASIL-Stroke patients (vs. CADASIL-non-Stroke; median [IQR] CCI: 5 [3–7] vs. 4 [2–7]; *p* < 0.001). CADASIL-Stroke patients (vs. CADASIL-non-Stroke) had a higher reported medication utilization for indications of anti-migraines (66.3% vs. 55.7%; *p* = 0.003), anti-depressants / anti-psychotics (55.3% vs. 46.7%; *p* = 0.018), and anti-thrombotics (53.7% vs. 29.3%; *p* < 0.001) as well as use of statins (57.0% vs. 36.0%; *p* < 0.001). No differences were observed with respect to vital sign measurements.

Reported mortality rates were not statistically different between the CADASIL-Stroke (4.9%) and CADASIL-non-Stroke (7.5%) sub-cohorts (*p* = 0.133). The median (IQR) reported age of death was 66 (59–76) years in the CADASIL-Stroke group, compared to 68 (63–71) years in the CADASIL-non-Stroke group.

### Sex-stratified differences in stroke burden and outcomes in CADASIL patients

**Figure 2:**
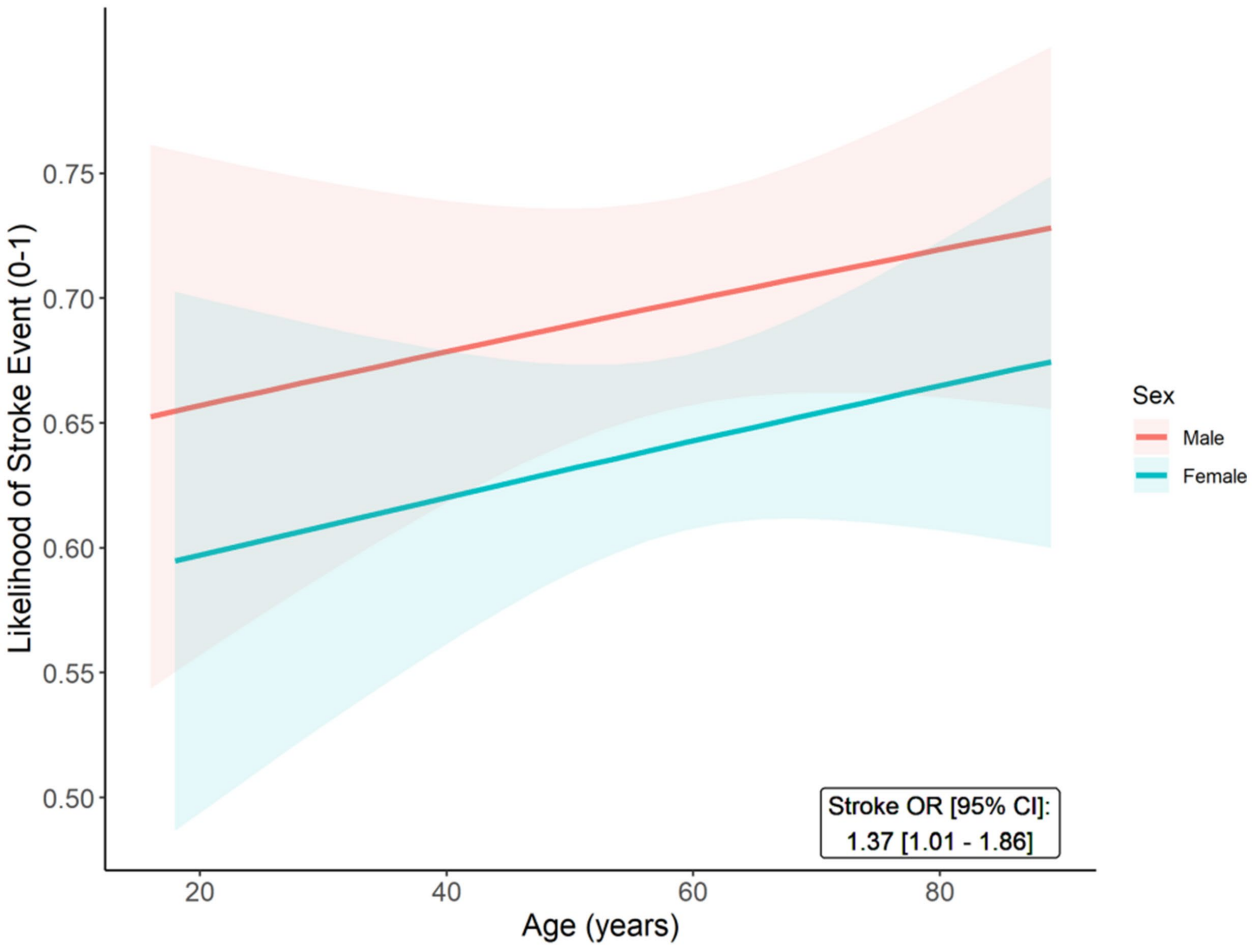
Predicted probabilities of stroke event (Y-axis) across age (X-axis) among male and female CADASIL patients. The age- and comorbidity-adjusted odds ratio (OR) and 95% confidence interval (95% CI) for stroke onset (male vs. female) is reported.

Sex-stratified analyses are reported in Table 1. Compared to females, an overall higher proportion of males experienced their first recorded stroke event before the age of 65 years (51.0% vs. 43.2%; *p* = 0.026). Likewise, the initial stroke event occurred at a younger age for males (vs. females), however this difference was not statistically significant (median [IQR] age in years: 56 (48–65) vs. 58 (49–67); *p* = 0.081). A similar, non-significant trend (*p* = 0.270) was observed in the Kaplan–Meier curves for cumulative incidence of stroke events over time, with males seemingly exhibiting a higher event probability during middle age (45–65 years); however, the difference in the curves converges after this period (Supplementary Figure S1). Proportionately, strokes (68.3% vs. 63.2%; *p* = 0.136) and, in particular, ischemic strokes (62.0% vs. 55.9%; *p* = 0.078) were non-significantly more frequent in males, compared to females. In age- and comorbidity-adjusted (migraine, epilepsy / seizure, mood disorders, cognitive impairment, hypertension, hyperlipidemia, diabetes) multivariable models, males had a higher likelihood of experiencing CADASIL associated stroke (OR: 1.37, 95% CI: 1.01–1.86). Estimates for the sex-stratified probabilities of a stroke event across the spectrum of age are depicted in Figure 2 and show that males have a consistently higher risk than females. Furthermore, migraines (OR: 1.63, 95% CI: 1.17–2.27), epilepsy / seizures (OR: 1.59, 95% CI: 1.03–2.44), cognitive impairment (OR: 1.74, 95% CI: 1.25–2.42), and hyperlipidemia (OR: 2.40, 95% CI: 1.69–3.40) were associated with stroke among CADASIL patients.

**Table 1:** Sex-stratified stroke onset and clinical outcomes in CADASIL patients.

	Male (*n* = 353)	Female (*n* = 560)	*p*-value
**Stroke**	241 (68.3)	354 (63.2)	0.136
Ischemic stroke (IS)	219 (62.0)	313 (55.9)	0.078
Intracerebral hemorrhage (ICH)	20 (5.7)	21 (3.8)	0.231
Subarachnoid hemorrhage (SAH)	6 (1.7)	4 (0.7)	0.286
Transient ischemic attack (TIA)	85 (24.1)	133 (23.8)	0.973
**Age at first stroke (years) – median (IQR)**	56 (48–65)	58 (49–67)	0.081
Range (years)	15–87	24–89	-
≤65	180 (51.0)	242 (43.2)	0.026
<18	1 (0.3)	0 (0)	0.816
18–30	3 (0.8)	9 (1.6)	0.497
31–50	75 (21.2)	90 (16.1)	0.059
51–65	101 (28.6)	143 (25.5)	0.344
>65	59 (16.7)	106 (18.9)	0.448
66–80	54 (15.3)	97 (17.3)	0.478
>80	5 (1.4)	9 (1.6)	1.000
NA	2 (0.6)	6 (1.1)	-
**Died**	32 (9.1)	21 (3.8)	0.001
Age at death (years) – median (IQR)	68 (63.8–70)	69 (62.5–72)	1.000
Range	53–89	21–89	-
Had Stroke	14 (4.0)	15 (2.7)	0.375

Excluding 1 patient with missing sex information. IQR, interquartile range.

**Figure 3:**
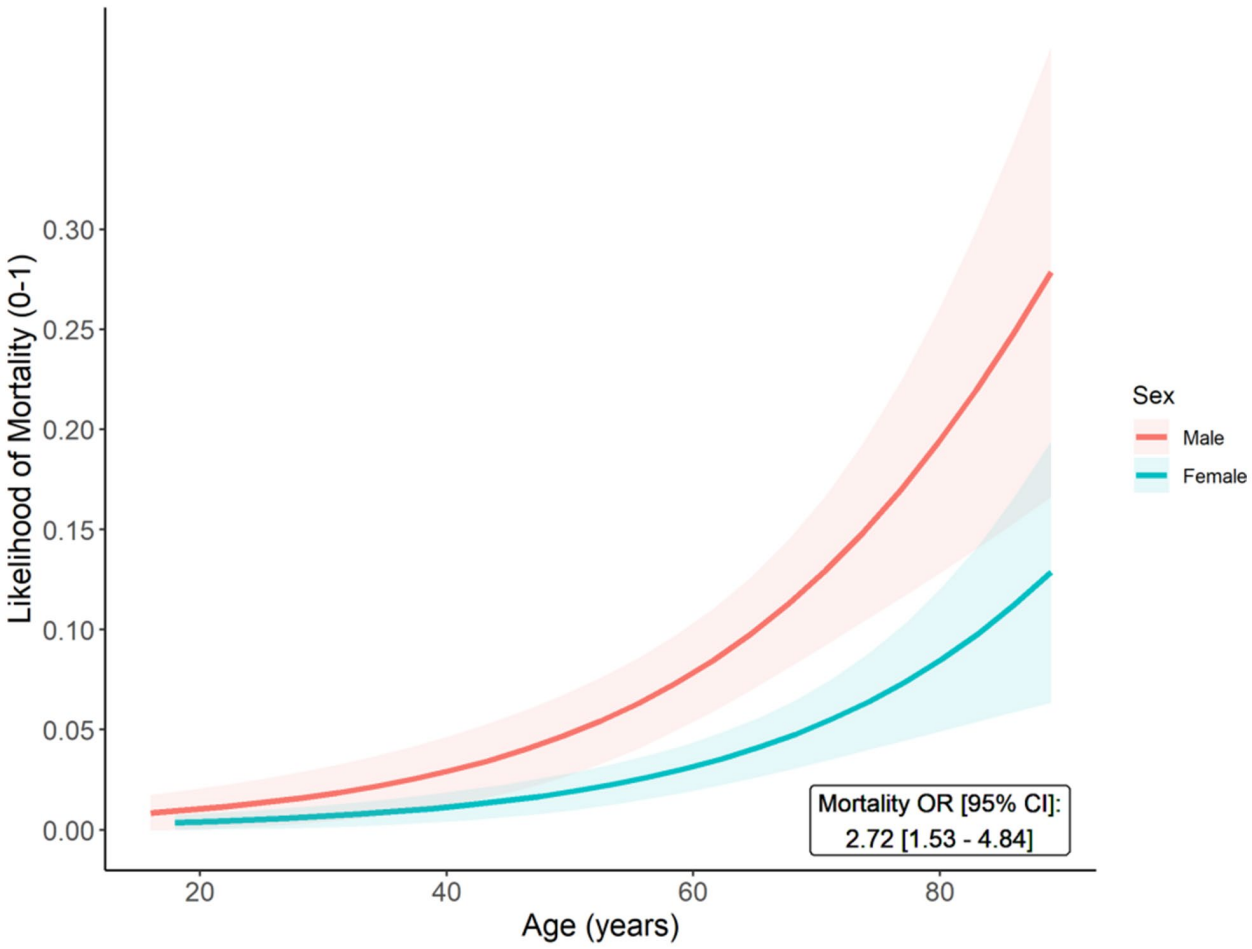
Predicted probabilities of mortality (Y-axis) across age (X-axis) among male and female CADASIL patients. The age- and stroke-adjusted odds ratio (OR) and 95% confidence interval (95% CI) for mortality (male vs. female) is reported.

Mortality rates were higher among male (vs. female) CADASIL patients (9.1% vs. 3.8%; *p* = 0.001). In addition, Kaplan–Meier curves demonstrate a significantly higher cumulative incidence of mortality for males, compared to females (*p* = 0.001) (Supplementary Figure S2). When evaluated using multivariable models, which were adjusted for age and prior stroke, this translated to males having a significantly higher likelihood of overall mortality (OR: 2.72, 95% CI: 1.53–4.84) (Figure 3). Although advanced age was associated with mortality (OR: 1.62, 95% CI: 1.29–2.03), stroke was not independently related to mortality (OR: 0.57, 95% CI: 0.32–1.01) in the adjusted models.

### Co-presenting and principal diagnoses and symptoms in CADASIL patients

Irrespective of documented stroke, we aggregated summary counts of frequently presenting diagnoses across historical clinical encounters (*n* = 51,376) for the overall CADASIL patient cohort from 2018 to 2020. The 20 most frequent co-presenting and principal diagnoses are shown in Supplementary Table S2. Among CADASIL-specific encounters in which CADASIL was documented as the principal diagnosis for the hospital encounter (*n* = 2,401), patients most often co-presented with hypertension (≥27.0%), hyperlipidemia (≥15.6%), and cerebral infarctions (≥14.5%); other common co-diagnoses included major depressive or anxiety disorders (≥14.7%) as well as headaches and migraines (≥11.0%).

## Discussion

We report the overt burden of cerebrovascular and neurological disease and outcomes among a large sample of CADASIL patients in the United States. We demonstrate higher associated risks of stroke events and mortality for male (vs. female) CADASIL patients.

Our characterization of the clinical features associated with CADASIL is consistent with prior reporting, with patients frequently presenting with cerebrovascular events (strokes), neurological conditions (migraines, cognitive impairment), neuro-psychiatric symptoms (mood disorders), and other vascular risk factors (hypertension, hyperlipidemia, diabetes) (14). However, specific rates for conditions varied across our cohort and that of other non-United States based studies. Although frequency of stroke events in our study (65%) were similar to prior reporting (61–62%), rates of conventional vascular risk factors were elevated in our United States cohort: hypertension (67% vs. 13–23%), hyperlipidemia (71% vs. 58%), and diabetes (30% vs. 10%) (4, 7). Conversely, in our study, respective rates for neurological conditions such as migraines (34%) and cognitive impairment (34%) fell in between estimates from other Asian (33 and 20%) and European (70 and 48%) based studies (4, 7).

To expand on previous reports, we additionally analyzed the clinical course and timing of stroke events in the registry data. Despite females representing a higher frequency of our CADASIL cohort, our findings are in line with prior (predominantly non-United States based) studies that indicate earlier onset of stroke and increased mortality in male CADASIL patients. In contrast to a previous retrospective study of 400 CADASIL patients in Germany, we found increased median ages (years) at time of initial documented stroke for both men and women: [56 (48–65) vs. 50.7 (48.2–53.1) and 58 (49–67) vs. 52.5 (50.0–54.9), respectively] (12). The authors of the German study also compared survival times to national life expectancy tables and found males – but not females – to have relatively shorter survival. Although survival was not extensively evaluated in our study, we did similarly observe males to have higher likelihoods of stroke onset and mortality. A more recent study from Scotland examining sex differences in CADASIL patients found estimates in age at initial stroke to lie in between that of the German study and our current analyses; nonetheless, the estimates were similarly disparate (median [IQR] age: males, 52 [45.3–58.7] vs. females, 57 [54.1–59.8]) (7). Although the direct causes for sex as a risk factor have not been established, growing evidence has suggested that differences in clinical severity attributed to CADASIL may be due to underlying higher degrees of cerebral atrophy and larger volumes of subcortical infarcts often observed in males, compared to females (16). In addition, variations in the signaling and metabolic pathways of sex hormones may also be involved and contribute to a neuroprotective role in females (14, 16). Lastly, differences in lifestyle-related risk factors (e.g., smoking, alcohol consumption), health management, and socio-economic factors may also be potential explanations (14, 15).

In our analyses, we found rates of anti-platelet and anti-coagulant use to vary, respectively, from 24.0 and 10.0% in CADASIL-non-Stroke patients to 48.2 and 13.9% in CADASIL-Stroke patients. Specifically, among CADASIL patients with any documented anti-thrombotic medication use, rates of hemorrhagic and ischemic stroke events were 4.3 and 76.8%, respectively. In the absence of a cure for CADASIL, treatment strategies typically focus on symptom management, prevention of further atherogenic events, and provision of standard acute stroke care (14, 18, 19). Although anti-platelet therapy (e.g., low-dose aspirin) has been recommended for symptomatic CADASIL patients, evidence has not been established as to its effectiveness for primary stroke prevention (18). Nonetheless, a recent retrospective study found no increased risk of ischemic or hemorrhagic stroke events among CADASIL patients who were administered anti-platelet medication (20). There is some guidance against the use of oral anti-coagulants due to the increased risk of bleeding in the brain (21). To the best of our knowledge, the successful use of tissue plasminogen activator (t-PA) for treatment in CADASIL patients has been reported in a single case report, with no sign of intracranial hemorrhage (22). In our data, t-PA administration was reported in only 10 (1.1%) CADASIL patients, of which 7 had a documented cerebrovascular event; however, none were hemorrhagic in nature. Future studies need to evaluate the safety and efficacy of thrombolytic therapies among CADASIL patients with acute stroke.

Limitations of our study include potential misclassification of CADASIL. Although genetic tests to confirm a CADASIL diagnosis have been conducted in the past decades, a distinct ICD-10 code characterizing the hereditary component of the disease was not established until the fourth quarter of 2018 (23). Prior to this, there was overlap in reporting of CADASIL with other cerebrovascular conditions (e.g., stroke, vascular dementia, epilepsy) and different monogenic cerebral small vessel diseases (24, 25). Although our data does not include detailed information on genetic tests for mutations in the *NOTCH3* gene, we expected the introduction of a distinct ICD-10 code to minimize overlap with other monogenic cerebral small vessel diseases or phenotypically similar conditions. Notably, a recent United States based, single-site study presented preliminary findings from a conference abstract in which potential CADASIL patients were identified by querying ICD-10 codes and EMR notes for keywords (26). Among the 750 patients identified, 150 were reviewed, of which 13% had a *NOTCH3* mutation and/or clinically diagnosed CADASIL. However, given that these reported estimates represented preliminary findings, detailed characterization from larger United States based cohorts are still necessary. Lastly, the research network includes shared data from multiple EMR systems. Consequently, differences in reporting across sites, anonymization protocols, and incomplete clinical and demographic information are inherent limitations of harmonized EMR databases.

Our findings underscore the need to further evaluate the biological pathways that predispose CADASIL patients to higher lifetime burden of stroke and other neurological conditions. Early screening and targeted treatment strategies may help mitigate the risk of poor outcomes and help patients/providers manage symptoms. Detailed phenotype and genotype characterization of CADASIL patients via cross-institutional registries will allow for analyses that can evaluate hypotheses regarding prevalence, genetic/environmental risks among subpopulations, treatments and outcomes.

## Data availability statement

The original contributions presented in the study are included in the article/Supplementary materials, further inquiries can be directed to the corresponding author.

## Ethics statement

Ethical review and approval was not required for the study on human participants in accordance with the local legislation and institutional requirements. The patients/participants provided their written informed consent to participate in this study.

## Author contributions

AP and FV were responsible for the study conception and design. AP performed the data acquisition and analysis. AP, TP, AB, JT, and FV completed interpretation of results and initial drafting of the manuscript. All authors contributed to the article and approved the submitted version.

## Conflict of interest

The authors declare that the research was conducted in the absence of any commercial or financial relationships that could be construed as a potential conflict of interest.

## Publisher’s note

All claims expressed in this article are solely those of the authors and do not necessarily represent those of their affiliated organizations, or those of the publisher, the editors and the reviewers. Any product that may be evaluated in this article, or claim that may be made by its manufacturer, is not guaranteed or endorsed by the publisher.
