# Supplementary material for: Lifelong cerebrovascular disease burden among CADASIL patients: analysis from a global health research network
Source: Front Neurol. 2023 Jul 14;14:1203985. doi: 10.3389/fneur.2023.1203985 (PMC10375407; doi:10.3389/fneur.2023.1203985)

Supplementary Material

Lifelong Cerebrovascular Disease Burden among CADASIL Patients: Analysis from a Global Health Research Network

Alan P. Pan MS[1], Thomas Potter PhD[2], Abdulaziz Bako PhD MBBS MPH[2], Jonika Tannous PhD[2], Sudha Seshadri MD[3], Louise D. McCullough MD PhD[4], Farhaan S. Vahidy PhD MBBS MPH[1,2,5*]

[1]Center for Health Data Science and Analytics, Houston Methodist, Houston, TX, USA
[2]Department of Neurosurgery, Houston Methodist, Houston, TX, USA
[3]Glenn Biggs Institute for Alzheimer's and Neurodegenerative Diseases, University of Texas Health Science Center, San Antonio, TX, USA
[4]Department of Neurology, McGovern Medical School, University of Texas Health Science Center, Houston, TX, USA
[5]Department of Population Health Sciences, Weill Cornell Medicine, New York, NY, USA*****

Correspondence:
Farhaan S. Vahidy, PhD MBBS MPH FAHA
fvahidy@houstonmethodist.org

Supplementary Table 1. Demographic, Comorbidity, and Clinical Outcome Characteristics in CADASIL Patients, by History of Stroke

	CADASIL Cohort (n = 914)	*CADASIL with Stroke (n = 596)*	*CADASIL without Stroke (n = 318)*	*P-Value*
Demographics and Social Characteristics				
Age at last follow-up or death (years) – mean (SD)	59.2 (13.6)	60.1 (12.8)	57.6 (14.9)	0.012
Median (IQR)	60 (50 – 69)	61 (51 – 69)	59 (48 – 69)	0.044
Female (vs. Male) – n (%)	560 (61.3)	354 (59.4)	206 (64.8)	0.129
Race – n (%)				
White	224 / 256 (87.5)	146 / 165 (88.5)	78 / 91 (85.7)	0.657
Black	30 / 256 (11.7)	18 / 165 (10.9)	12 / 91 (13.2)	0.734
Asian	2 / 256 (0.8)	1 / 165 (0.6)	1 / 91 (1.1)	1.000
NA	658 (72.0)	431 (72.3)	227 (71.4)	-
Ethnicity – n (%)				
Hispanic	29 / 291 (10.0)	20 / 190 (10.5)	9 / 101 (8.9)	0.816
Non-Hispanic	262 / 291 (90.0)	170 / 190 (89.5)	92 / 101 (91.1)	0.816
NA	623 (68.2)	406 (68.1)	217 (68.2)	-
US geographic area (3-digit ZIP code)				
0 (~New England)	51 (5.6)	25 (4.2)	26 (8.2)	0.019
1 (~Middle Atlantic)	105 (11.5)	69 (11.6)	36 (11.3)	0.995
2 (~South Atlantic)	60 (6.6)	43 (7.2)	17 (5.3)	0.344
3 (East South Central)	114 (12.5)	72 (12.1)	42 (13.2)	0.699
4 (~East North Central)	103 (11.3)	79 (13.3)	24 (7.5)	0.013
5 (~West North Central)	60 (6.6)	44 (7.4)	16 (5.0)	0.220
6 (~West North Central)	57 (6.2)	32 (5.4)	25 (7.9)	0.180
7 (West South Central)	58 (6.3)	27 (4.5)	31 (9.7)	0.003
8 (Mountain)	48 (5.3)	28 (4.7)	20 (6.3)	0.383
9 (Pacific)	114 (12.5)	73 (12.2)	41 (12.9)	0.860
NA	144 (15.8)	104 (17.4)	40 (12.6)	0.067
Comorbidities and Co-existing Conditions				
**Neurological**				
Migraine	314 (34.4)	219 (36.7)	95 (29.9)	0.044
Migraine with aura	105 (11.5)	76 (12.8)	29 (9.2)	0.126
Cognitive impairment	308 (33.7)	231 (38.8)	77 (24.2)	<0.001
Epilepsy / Seizures	148 (16.2)	111 (18.6)	37 (11.6)	0.008
White matter disease	126 (13.8)	91 (15.3)	35 (11.0)	0.093
Cerebral amyloid angiopathy	8 (0.9)	6 (1.0)	2 (0.6)	0.833
Leukoencephalopathy				
Multiple sclerosis	90 (9.8)	57 (9.6)	33 (10.4)	0.782
Parkinson’s disease	40 (4.4)	31 (5.2)	9 (2.8)	0.134
**Stroke**	596 (65.2)	596 (100)	-	-
Ischemic stroke (IS)	533 (58.3)	533 (89.4)	-	-
Lacunar infarction	-	28 (4.7)	-	-
Intracerebral hemorrhage (ICH)	41 (4.5)	41 (6.9)	-	-
Subarachnoid hemorrhage (SAH)	10 (1.1)	10 (1.7)	-	-
Transient ischemic attack (TIA)	218 (23.9)	218 (36.6)	-	-
Age at first stroke (years) – median (IQR)	-	58 (48 – 66)	-	-
Range (years)	-	15 – 89	-	-

<18	-	1 (0.2)	-	-
18 – 30	-	12 (2.0)	-	-
31 – 50	-	166 (27.9)	-	-
51 – 65	-	244 (40.9)	-	-
66 – 80	-	151 (25.3)	-	-
>80	-	14 (2.3)	-	-
NA	-	8 (1.3)	-	-
NIH Stroke Scale – median (IQR)	-	3.0 (1.0 – 6.8)	-	-
NA	-	530 (88.9)	-	-
Glasgow Coma Scale				
3 – 8	1	0	1	-
9 – 12	5	4	1	-
13 – 15	6	5	1	-
Other coma	0	0	0	-
NA	902	587	315	-
**Neuro-psychiatric**				
Mood (affective) disorders	445 (48.7)	315 (52.9)	130 (40.9)	0.001
Symptoms involving emotion	65 (7.1)	47 (7.9)	18 (5.7)	0.266
**Other**				
Hypertension	610 (66.7)	418 (70.1)	192 (60.4)	0.004
Hyperlipidemia	648 (70.9)	465 (78.0)	183 (57.5)	<0.001
Atrial fibrillation	79 (8.6)	61 (10.2)	18 (5.7)	0.026
**Charlson Comorbidity Index (CCI) Score – median (IQR)**	5 (3 – 7)	5 (3 – 7)	4 (2 – 7)	<0.001
Myocardial infarction	69 (7.5)	45 (7.6)	24 (7.5)	1.000
Congestive heart failure	110 (12.0)	69 (11.6)	41 (12.9)	0.634
Peripheral vascular disease	291 (31.8)	216 (36.2)	75 (23.6)	<0.001
Dementia	261 (28.6)	200 (33.6)	61 (19.2)	<0.001
Chronic obstructive pulmonary disease	236 (25.8)	152 (25.5)	84 (26.4)	0.825
Connective tissue disease	72 (7.9)	43 (7.2)	29 (9.1)	0.374
Peptic ulcer	40 (4.4)	27 (4.5)	13 (4.1)	0.888
Liver disease (mild)	111 (12.1)	74 (12.4)	37 (11.6)	0.812
Liver disease (moderate to severe)	11 (1.2)	6 (1.0)	5 (1.6)	0.668
Diabetes w/o complications	272 (29.8)	193 (32.4)	79 (24.8)	0.022
Diabetes with complications	131 (14.3)	90 (15.1)	41 (12.9)	0.419
Hemiplegia	157 (17.2)	139 (23.3)	18 (5.7)	<0.001
Chronic kidney disease (mild to moderate)	147 (16.1)	95 (15.9)	52 (16.4)	0.946
Solid tumor (localized)	121 (13.2)	68 (11.4)	53 (16.7)	0.033
Solid tumor (metastatic)	25 (2.7)	13 (2.2)	12 (3.8)	0.233
AIDS / HIV	2 (0.2)	2 (0.3)	0 (0)	0.771
Medications / Therapeutics				
Anti-migraine	548 (62.6)	381 / 575 (66.3)	167 / 300 (55.7)	0.003
Vasoconstrictors	106 (12.1)	65 / 575 (11.3)	41 / 300 (13.7)	0.364
Anti-depressants / Anti-psychotics	458 (52.3)	318 / 575 (55.3)	140 / 300 (46.7)	0.018
Anti-thrombotics	397 (45.4)	309 / 575 (53.7)	88 / 300 (29.3)	<0.001
Anti-platelets	349 (39.9)	277 / 575 (48.2)	72 / 300 (24.0)	<0.001
Novel oral anti-coagulants (NOACs)	110 (12.6)	80 / 575 (13.9)	30 / 300 (10.0)	0.121
Anti-hypertensives	363 (41.5)	252 / 575 (43.8)	111 / 300 (37.0)	0.061
Statins	436 (49.8)	328 / 575 (57.0)	108 / 300 (36.0)	<0.001
Anti-convulsants	244 (27.9)	169 / 575 (29.4)	75 / 300 (25.0)	0.195

Donepezil	68 (7.8)	57 / 575 (9.9)	11 / 300 (3.7)	0.002
Tissue plasminogen activator (t-PA)	10 (1.1)	7 (1.2)	3 (0.9)	1.000
Vital Signs – mean (sd)				
Body mass index (BMI)	29.5 (6.0)	29.7 (6.1)	29.1 (5.7)	0.323
Systolic blood pressure (SBP)	124.8 (12.6)	125.0 (12.4)	124.3 (13.1)	0.629
Diastolic blood pressure (DBP)	76.3 (7.2)	76.3 (7.1)	76.2 (7.4)	0.954
Temperature (°F)	97.9 (0.6)	97.9 (0.5)	98.0 (0.7)	0.764
Respiratory rate (breaths / min)	13.9 (6.0)	14.0 (6.2)	13.8 (5.9)	0.855
Heart rate (beats / min)	73.1 (18.6)	72.2 (18.1)	74.9 (19.4)	0.197
Outcomes				
Died	53 (5.8)	29 (4.9)	24 (7.5)	0.133
Age at death (years) – median (IQR)	68 (60 – 73)	68 (63 – 71)	66 (59 – 76)	0.592
Range	21 – 89	46 – 89	21 – 88	-

SD: standard deviation, IQR: interquartile range, AIDS: acquired immunodeficiency syndrome, HIV: human immunodeficiency virus
Missing: Age (n=12), Sex (n=1), Race (n=658), Ethnicity (n=653), Medications (n=39)

Supplementary Table 2. Frequent Principal and Co-Presenting Diagnoses and Symptoms in CADASIL Patients. (A) represents hospital encounters in which CADASIL was documented as the principal diagnosis and reason for visit.

Co-presenting Diagnoses at CADASIL-Specific Clinical Encounters (n = 2,401)	Frequency (%)
Essential (primary) hypertension	649 (27.0)
Hyperlipidemia, unspecified	374 (15.6)
Cerebral infarction, unspecified	347 (14.5)
Personal history of transient ischemic attack and cerebral infarction without residual deficits	236 (9.8)
Major depressive disorder, single episode, unspecified	178 (7.4)
Anxiety disorder, unspecified	175 (7.3)
Type 2 diabetes mellitus without complications	158 (6.6)
Hypothyroidism, unspecified	153 (6.4)
Gastro-esophageal reflux disease without esophagitis	152 (6.3)
Acute kidney failure, unspecified	144 (6.0)
Other long term (current) drug therapy	144 (6.0)
Unsteadiness on feet	139 (5.8)
Headache	134 (5.6)
Migraine, unspecified, not intractable, without status migrainosus	129 (5.4)
Unspecified convulsions	125 (5.2)
Dementia in other diseases classified elsewhere with behavioral disturbance	123 (5.1)
Weakness	123 (5.1)
Long term (current) use of aspirin	122 (5.1)
Obstructive sleep apnea (adult) (pediatric)	111 (4.6)
Muscle weakness (generalized)	110 (4.6)
Description of diagnoses taken from International Classification of Diseases, Ninth and Tenth Revision (ICD-9 and ICD-10)	

Supplementary Figure 1. Kaplan-Meier curves for sex-stratified cumulative incidence of stroke onset (Y-axis) across age (X-axis).

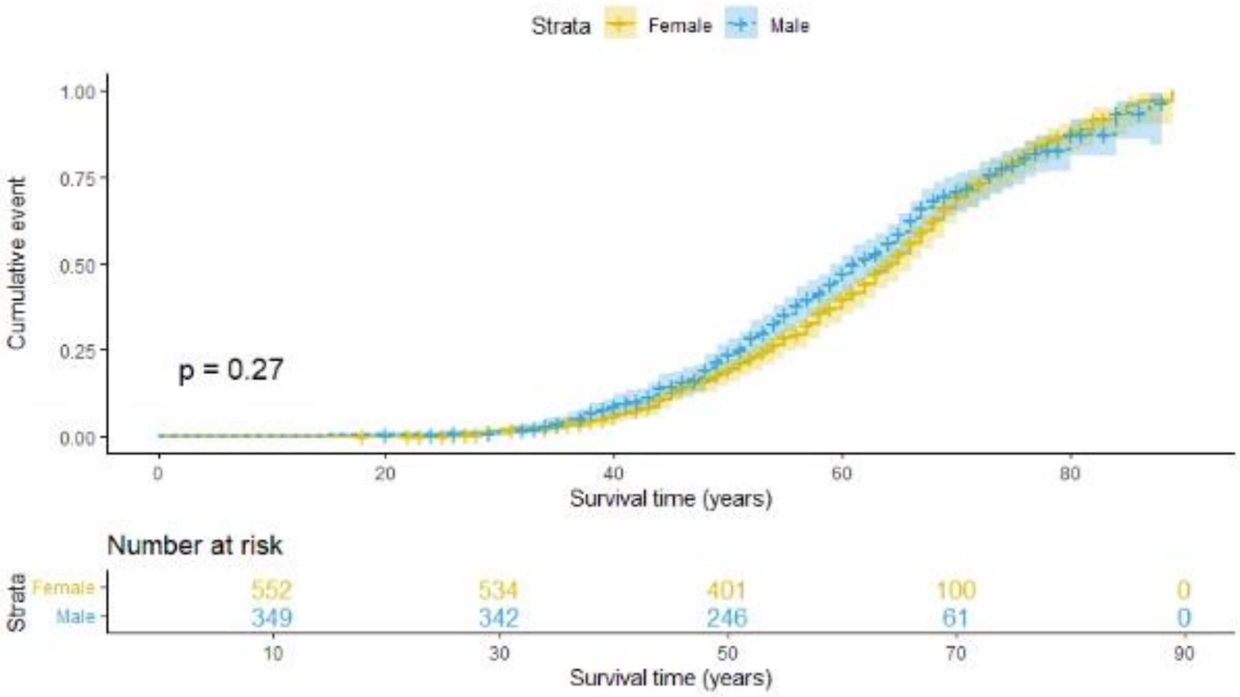

Supplementary Figure 2. Kaplan-Meier curves for sex-stratified cumulative incidence of mortality (Y-axis) across age (X-axis).

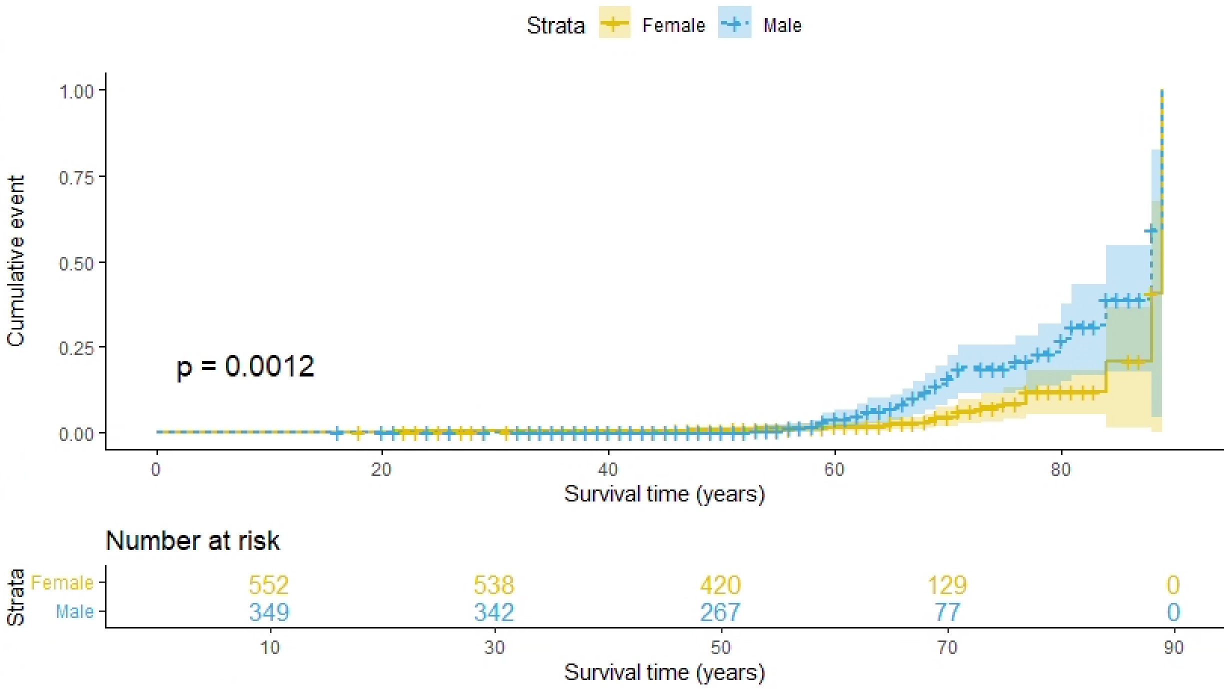

Supplement: Supplementary file 1 [file Data_Sheet_1.PDF]
